# Role of *mprF1* and *mprF2* in the Pathogenicity of *Enterococcus faecalis*

**DOI:** 10.1371/journal.pone.0038458

**Published:** 2012-06-18

**Authors:** Yinyin Bao, Tuerkan Sakinc, Diana Laverde, Dominique Wobser, Abdellah Benachour, Christian Theilacker, Axel Hartke, Johannes Huebner

**Affiliations:** 1 Division of Infectious Diseases, Department of Medicine, University Hospital Freiburg, Freiburg, Germany; 2 EA4655 U2RM Stress/Virulence, Université de Caen Basse-Normandie, Caen, France; University of Illinois at Chicago College of Medicine, United States of America

## Abstract

**Background:**

*Enterococcus faecalis* is one of the leading causes of nosocomial infections. Due to its innate and acquired resistance to most antibiotics, identification of new targets for antimicrobial treatment of *E. faecalis* is a high priority. The multiple peptide resistance factor MprF, which was first described in *Staphylococcus aureus,* modifies phosphatidylglycerol with lysin and reduces the negative charge of the membrane, thus increasing resistance to cationic antimicrobial peptides. We studied the effect of *mprF* in *E. faecalis* regarding influence on bacterial physiology and virulence.

**Results:**

Two putative *mprF* paralogs (*mprF1* and *mprF2*) were identified in *E. faecalis* by BLAST search using the well-described *S. aureus* gene as a lead. Two deletion mutants in *E. faecalis* 12030 were created by homologous recombination. Analysis of both mutants by thin-layer chromatography showed that inactivation of *mprF*2 abolishes the synthesis of three distinct amino-phosphatidylglycerols (PGs). In contrast, deletion of *mprF1* did not interfere with the biosynthesis of amino-PG. Inactivation of *mprF*2 increased susceptibility against several antimicrobial peptides and resulted in a 42% increased biofilm formation compared to wild-type *mprF*. However, resistance to opsonic killing was increased in the mutant, while virulence in a mouse bacteremia model was unchanged.

**Conclusion:**

Our data suggest that only *mprF2* is involved in the aminoacylation of PG in enterococci, and is probably responsible for synthesis of Lys-PG, Ala-PG, and Arg-PG, while *mprF1* does not seem to have a role in aminoacylation. As in other Gram-positive pathogens, aminoacylation through MprF2 increases resistance against cationic antimicrobial peptides. Unlike *mprF* found in other bacteria, *mprF*2 does not seem to be a major virulence factor in enterococci.

## Introduction

*Enterococcus faecalis* is part of the normal flora in the gastro-intestinal tract of humans and animals. Some strains have been used as probiotics, whereas others are the cause of serious and sometimes life-threatening infections [1], [2]. Due to its innate and acquired resistance to most clinically used antibiotics, treatment of serious infections by enterococci is often limited in effect and sometimes impossible [3].

The main lipid constituents of bacterial membranes are phospholipids. The two major bacterial phospholipids are phosphatidylglycerol (PG) and diphosphatidylglycerol (DPG). Their head groups are negatively charged, thereby imparting anionic properties to the membrane surface. Many bacteria can modify negatively charged lipids with positively charged substituents, such as lysine, to form lysyl-phosphatidylglycerol (Lys-PG), reducing the negative net charge of the membrane surface. Lys-PG is synthesized by the integral membrane protein MprF, which transfers a lysyl group from lysyl-tRNA to PG and subsequently translocates Lys-PG from the inner to the outer leaflet of the cytoplasmic membrane [4]. The reduced negative net charge of the cell membrane leads to the repulsion of cationic peptides, decreasing the sensitivity against these peptides [5]. In addition, this mechanism also bestows *S. aureus* with resistance to cationic antibiotics such as daptomycin, vancomycin [6], and gentamicin [7]. Furthermore, MprF is also considered a virulence factor, since it allows bacteria to evade neutrophil killing and enhances the virulence of *S. aureus* in mice [8].

In addition to *S. aureus,* the *mprF* gene is also present in the genomes of several other clinically important pathogens, such as *Mycobacterium tuberculosis, Pseudomonas aeroginosa*, *Listeria monocytogenes*, and *E. faecalis* [9]. Two putative *mprF* genes were found in *E. faecalis*, *Enterococcus faecium*, and several other gram-positive species [10].

In the present study, we used targeted mutagenesis to inactivate the two *mprF* paralogs in *E. faecalis* to characterize the resulting changes in cell wall lipids and to investigate the contribution of phosphatidylglycerol aminoacylation to resistance, biofilm formation, and virulence.

## Results

### Identification and Sequence Analysis of Two *mprF* Paralogs in *E. faecalis*

Blast-search analysis of the genome sequences of *E. faecalis* V583 [11] and *E. faecalis* 12030 (unpublished results) identified genes with significant homology in these two organisms: *EF_0031* and *EF_1027*, sharing 24% and 31% amino-acid identity (respectively) with the *mprF* gene of *S. aureus* (accession number ADJ67256.1). According to homologies with genes characterized by Roy and Ibba, we provisionally named these two genes *mprF1 (EF_0031)* and *mprF2* (*EF_1027*) [9], [10]. The MprF protein of *S. aureus* consists of two functional domains [4]. The hydrophilic C-terminus demonstrates aminoacyl phosphatidylglycerol (PG)-synthase activity, and the hydrophobic N-terminus functions as a flippase, transferring aminoacyl-PG from the inner to the outer leaflet of the cell membrane. The homology of the two domains in *S. aureus* with the two *mprF* genes in *E. faecalis* 12030 was assessed: The N-terminal part (corresponding to the flippase) of *mprF1* and *mprF2* shows 24% and 31% identity with *S. aureus,* respectively. The C-terminus (synthase) of *mprF1* and *mprF2* demonstrates 29% and 40% identity (respectively) with the synthase of the *S. aureus* gene.

### Growth Kinetics

The mutants 12030*ΔmprF1*, 12030*ΔmprF2*, and the respective complemented strains were no different than the wild-type (WT) regarding growth kinetics in broth culture (data not shown).

### Only Deletion of *mprF2* Leads to Complete Loss of Amino-phospholipids

**Figure 1:**
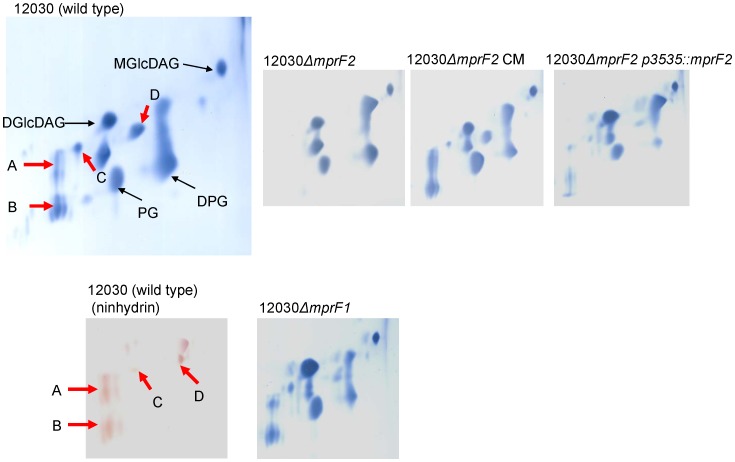
Lipid analysis of the wild-type and its mutants by two dimensional thin-layer chromatography. Cell membrane total lipid extracts from *E. faecalis* 12030 (wild type), 12030*ΔmprF1* (*EF_0031*), 12030*ΔmprF2* (*EF_1027*), 12030*ΔmprF2* CM (knock-in complementation), 12030*ΔmprF2 p3535::mprF2*. Lipids were separated using a solvent system of CHCl_3_/MeOH/H_2_O(65∶15:2, v/v/v) in the first dimension, and CHCl_3_/MeOH/Acetic acid/H_2_O (80/12/15/4, v/v/v) in the second dimension. Aminophospholipids were visualized with molybdenum stain solution, 12030 (wild-type) was also stained with ninhydrin. PG – phosphatidylgylcerol, DPG – diphosphaditylglyerol, DGlcDAG – diglycosyldiacylglycerol. MGlcDAG – monoglycosyldiacylglycerol.

To confirm whether these two putative enterococcal *mprF* genes function similarly to the *mprF* of *S. aureus*, we compared the membrane lipid composition of the wild-type and mutant strains by two-dimensional thin-layer chromatography (2D-TLC). Four prominent ninhydrin-positive spots present in the wild-type were completely absent from the *mprF2* mutant, but reappeared upon complementation by knocking-in of the wild-type allele into the chromosome of the mutant, and by expression of *mprF2* in trans on plasmid *pMSP3535::mprF2* (see Figure 1). All spots mentioned above (A–D) were stained with molybdenum blue and ninhydrin, indicating that they represent amino-phospholipids. In *E. faecalis*, PG is usually acylated by two molecules of Lysin [12], [13]. Spots A and B migrate similarly to the two Lys-PG spots identified by Peschel and colleagues [8]. Spots C and D migrate similarly to Arg-PG [10] and Ala-PG [9], respectively. Deletion of *mprF1* did not seem to have an effect on amino-phospholipids, because in the deletion mutant 12030*ΔmprF1,* all spots were identical to the wild type.

### *mprF2* is Involved in Resistance Against Antimicrobial Peptides

The absence of Lys-PG from the membrane of *S. aureus* decreased the minimal inhibitory concentration (MIC) of certain cationic antimicrobial peptides (CAMP) [8]. The cationic antimicrobial peptides (CAMP) colistin, nisin, HBD-3, and polymyxin B were tested against the parental strain *E. faecalis* 12030 and its isogenic mutants. As shown in Table 1, 12030*ΔmprF2* showed a 2-fold decreased MIC against colistin, a 4-fold decreased MIC against polymyxin B and nisin, and a >4-fold decreased MIC against HBD-3 compared to the wild-type strain. Complementation of *mprF2* (12030*ΔmprF2* knock-in) completely restored the wild-type phenotype. In contrast, no difference in the sensitivity of the deletion mutant 12030*ΔmprF1* and the tested CAMPs was noted. E-test showed that the *E. faecalis* wild-type strain and mutants tested in this study were not significantly different in their sensitivity to daptomycin. The MICs of *E. faecalis* 12030, 12030*ΔmprF1*, 12030*ΔmprF2*, and 12030*ΔmprF2* CM (knock-in), were 0.19 mg/l, 0.25 mg/l, 0.25 mg/l, and 0.25 mg/l, respectively.

**Table 1:** Activities of cationic antimicrobial peptides against the *E. faecalis* 12030 wild type, the deletion mutants and the complemented strain.

Strains	Minimal inhibotory concentration (µg/ml)			
	Colistin	Polymyxin B	Nisin	HBD-3
***E. faecalis*** ** 12030**	4096	1024	4	>512
***E. faecalis*** ** 12030 ** ***ΔmprF1***	4096	1024	4	>512
***E. faecalis*** ** 12030 ** ***ΔmprF2***	2048	256	1	128
***E. faecalis*** ** 12030 ** ***ΔmprF2*** **CM**	4096	1024	4	>512

### MprF2 is Involved in Biofilm Formation and eDNA Release

**Figure 2:**
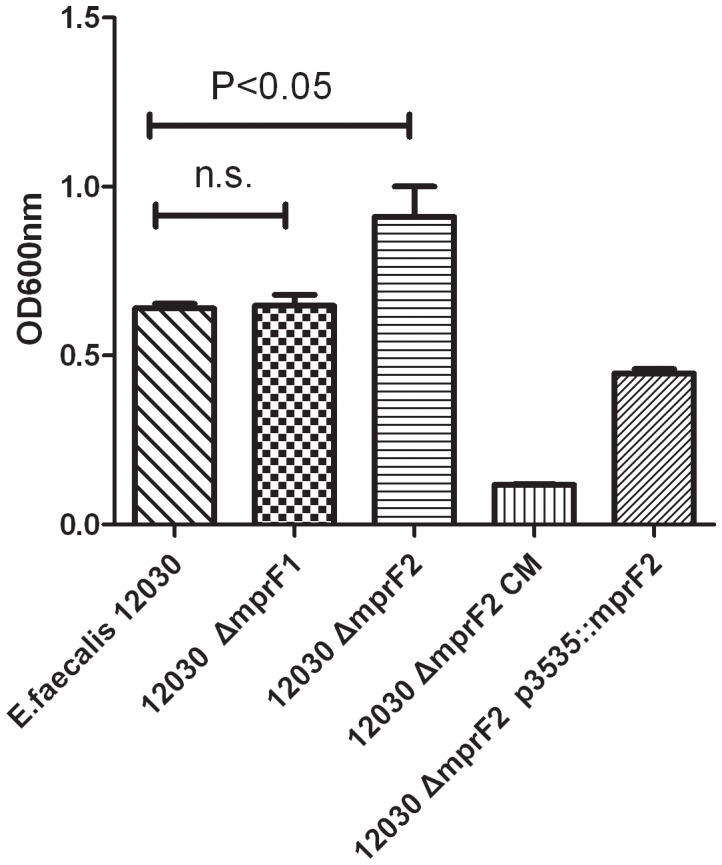
Biofilm production of the wild type and its derivate mutants. *E. faecalis* 12030, mutants 12030*ΔmprF2*1 and 12030*ΔmprF2* and complemented strains 12030*ΔmprF2* CM and 12030*ΔmprF2 P3535::mprF2* were cultivated in TSB media supplemented with 1% glucose. Bacteria were incubated for 18 h, unbound cells were removed by washing of the plates with buffer, and biofilm was stained with crystal violet. Error bars represent standard error of the mean.

**Figure 3:**
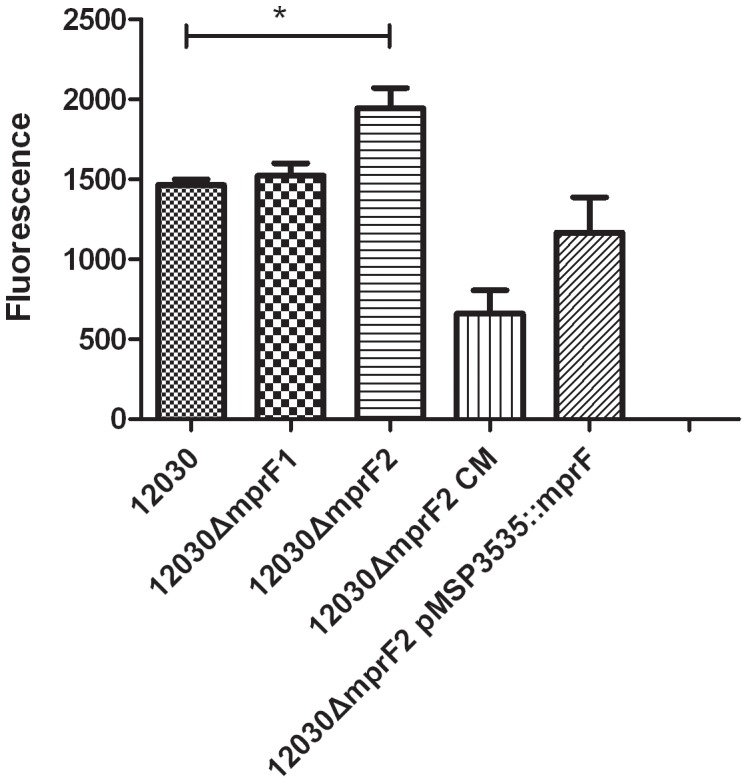
Measurement of eDNA in biofilms. All strains (12030, 12030*ΔmprF1*, 12030*ΔmprF2*, 12030*ΔmprF2* CM, 12030*ΔmprF2* pMSP3535::mprF2) were cultivated overnight in TSB at 37°C and measured with excitation wavelength at 485 nm and emission wavelength at 535 nm. *indicates statistical significance at p<0.05.

We showed previously that lack of D-alanine esters on teichoic acids leads to a decrease in biofilm formation, probably due to the increase in net charge of the bacterial cell surface [14]. Inactivation of MprF2 is predicted to increase the net negative charge on bacterial cells. We compared biofilm formation on polystyrene surfaces by the wild-type strain 12030 and its isogenic mutants, 12030*ΔmprF2* CM (knock-in) and 12030*ΔmprF2* p3535::*mprF2*. Surprisingly, the mutant 12030*ΔmprF2* produced significantly more biofilm than the wild type. Complementation by knock-in of *mprF2* (12030*ΔmprF2* CM knock-in) decreased the production of biofilm below wild-type levels, while biofilm production after complementation with a plasmid in trans (without induction by nisin) did not differ from the wild-type strain. The deletion mutant 12030*ΔmprF1* and the wild type produced similar amounts of biofilm (Figure 2). As shown in Figure 3 mutant 12030*ΔmprF2* released significantly more eDNA than the wild type and the mutant 12030*ΔmprF1*. The strain complemented by knock-in (12030*ΔmprF2* CM) produced significantly less eDNA biofilm than the wild type, while the mutant complemented by plasmid partially restored eDNA levels compared to the wild type.

### Triton X-100-induced Autolysis

Mechanisms affecting the modification of the membrane net charge of the peptidoglycan structure may play a role in the modulation of autolysin activity and thus may have an impact on bacterial autolysis. Therefore we evaluated the effect of autolysis by Triton-X100 on *E. faecalis* 12030*ΔmprF2* and the wild-type strain. No significant difference was found between them (data not shown), suggesting that *mprF2* has no obvious effect on autolysis in *E. faecalis*.

### The *mprF2* Mutant is More Resistant to Opsonophagocytic Killing than the Wild-type Strain

**Figure 4:**
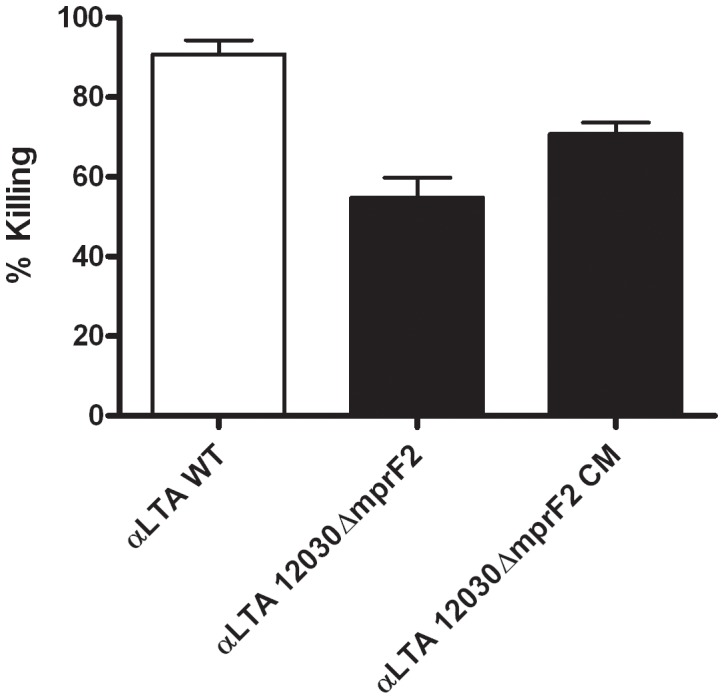
Effect of the deletion of MprF2 on resistance to opsonophagocytosis. Opsonophagocytic killing of the wild type (12030), 12030*ΔmprF2* and 12030*ΔmprF2* CM with serum against anti-LTA (serum-dilution of 1∶1,200).

Opsonophagocytic killing of *E. faecalis* 12030 wild-type and mutant 12030*ΔmprF2* was compared using log-phase-grown bacteria that were opsonized with rabbit complement in conjunction with antibodies against *E. faecalis* LTA [15]. Subsequently, numbers of surviving bacteria were determined. The *mprF2* mutant was killed significantly less than the wild type, and complementation by knock-in partially restored the killing to wild-type levels (Fig. 4). Opsonophagocytic killing in the presence of PMNs and complement alone did not differ between 12030*ΔmprF2* and the wild-type strain (data not shown).

### Mouse Bacteremia Model

**Figure 5:**
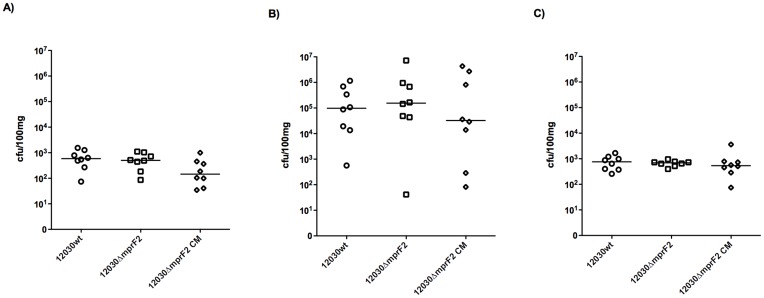
Effect of the deletion of the MprF2 on virulence in mice. 1.5×10^8^ CFU bacteria were injected in the tail vein of mice. Animals were sacrificed after 48 h, and colony counts were enumerated in liver, kidney and spleen.

Virulence of the *mprF2* mutant was assessed in a mouse bacteremia model as described previously [16]. The number of bacteria recovered from the liver, kidney, and spleen of mice infected with the *mprF2* mutant was not significantly different from those recovered from animals infected with wild-type (Figure 5).

## Discussion

Bacteria modulate the electrostatic properties of their cell envelope to protect themselves against the innate defense systems of the host, especially antimicrobial peptides [5]. Two different mechanisms that reduce the negative net charge of the bacterial cell wall have been identified. The first is modification of teichoic acid with D-alanine through the *dlt* operon (*dltABCD*) [17]. Inactivation of genes within this operon causes complete absence or reduction of teichoic acid D-alanine esters. This results in a higher negative net charge on the bacterial surface, because D-alanine esters neutralize the negative charge of teichoic acids [14]. The second charge-reducing mechanism is modification of membrane lipid phosphatidylglycerol (PG) with positively or neutrally charged amino-groups by *mprF*(multiple peptides resistance factor) [8], sometimes also described as aminoacylphosphatidylglycerol synthases (aaPGSs) [10].

The earliest report described the modification of PG with lysine in *S. aureus* [8]. However, It has long been known that certain bacteria produce Ala-PG (e.g., *Pseudomonas aeroginosa* [18]) instead of Lys-PG, while others produce both Lys-PG and Ala-PG (e.g., *Clostridium perfringens, Bacillus subtilis, and Enterococcus faecium;* Roy & Ibba 2009 [19]). Although Ala-PG has a neutral net charge, this modification has been demonstrated to increase bacterial resistance to certain CAMPs [18]. These observations suggest that *mprF-*mediated CAMPs resistance not only decreases the charge of the membrane but probably also modulates some biophysical properties of the membrane, such as fluidity and permeability [20].

The presence of two putative *mprF* paralogs in *E. faecium* DO was previously reported by Roy and Ibba [9], [10], and we confirmed this finding using translated BLAST (tblastn) of the well-characterized *mprF* gene of *S. aureus* against the genome of *E. faecalis* 12030 [21] and V583 [11]. Two genes were identified, with one (*EF_1027*) showing a higher homology than the other (*EF_0031*). Comparing these two putative *mprF* genes with the two aaPGs described by Roy and Ibba [10] showed that *mprF1* (*EF_0031*) and *mprF2* (*EF_1027*) share 57% and 62% identities of amino acids with the sequences of aaPGs1 and aaPGs2, respectively.

Lipid analysis of the deletion mutants in genes *mprF1* and *mprF2* in *E. faecalis* 12030 indicated that mutant *ΔmprF2* lacks aminoacyl phosphatidylglycerol, whereas no difference in lipid composition was seen between the mutant *ΔmprF1* and the wild type. To confirm these findings, we analyzed the composition of phospholipids of insertion mutants of *mprF1* and *mprF2* in a second strain, *E. faecalis* V583. Insertional inactivation of these genes in *E. faecalis* V583 produced the same phenotype and aminophospholipid patterns as noted for *E. faecalis* 12030 (data not shown). Therefore, we assume that *mprF1* (*EF_0031*) is not involved in the aminoacylatylation of PG, while *mprF2* (*EF_1027*) seems to be the only functional *mprF* gene in *E. faecalis*.

Roy and Ibba studied the aminoacylation of recombinantly expressed MprF1 and MprF2 from *Clostridium perfringens* in *E. coli*. They found that MprF1 was specific for Ala-PG, while only membrane extracts of *E. coli* expressing MprF2 were able to aminoacylate PG with Lys [9]. They also found that aaPGS1 from *E. faecium* DO (corresponding to MprF1) cannot use Lys-tRNA^Lys^ and Arg-tRNA^ Arg^ as aminoacyl group donors; however, results for Ala-PG were not reported. Using aaPGS2 of *E. faecium* and Lys-tRNA^Lys^, Ala-tRNA^Ala^, and Arg-tRNA^ Arg^ as donors, the final products Lys-PG, Ala-PG, and Arg-PG could be isolated from the of *E. coli* membranes [10].

Similar to previous reports [4], in *E. faecalis* 12030 we found that deletion of both *mprF* genes has no effect on growth (data not shown). Also similar to previously published results in *S. aureus* and other species, we found that the mutant 12030*ΔmprF2* (*EF_1027*) displayed a decrease in the MIC against nisin, colistin, polymyxin B, and HBD-3. Inactivation of MprF2 in *E. faecalis* had a lesser effect on the MIC of nisin (4-fold difference) than in *S. aureus*, where mutation of MprF resulted in a 28-fold drop of the MIC for this CAMP [8]. In contrast to *S. aureus,* however, there was no effect on daptomycin resistance. Interestingly, two recent reports identified other mutations in the synthetic pathway of phospholipids (i.e., diphosphatidylglycerol synthesis) being involved in daptomycin resistance of enterococci [22], [23], and this mechanism may be more important for resistance against the above-mentioned antimicrobial peptides. Our results suggest that Lys-PG, Ala-PG, and Arg-PG are probably not major factors involved in resistance to colistin, polymyxin B, nisin, or HBD-3 in enterococci. A *Bacillus anthracis mprF* mutant showed hyper-susceptibility to certain CAMPs (e.g., protamine, HNP-1, and LL-37) but exhibited only weak or no change in resistance to nisin [24], suggesting that MprF may be an important resistance mechanism for some CAMPs, but not for others. In *S. aureus* it has been reported that, depending on the individual strain, MprF may increase [25] or decrease [6] resistance to vancomycin, or may have no effect [26], with the respective phenotype probably being dependent on the genetic background of the isolate [6]. A great variety of additional resistance mechanisms and regulators are used by bacteria to circumvent the action of CAMPs [27]–[29].

It has been reported that in certain species (such as *Bacillus subtilis*, *Lactococcus lactis*, and *Streptococcus pyogenes*) the *dlt* operon affects autolysis by incorporation of D-Ala into lipoteichoic acids. Decreasing the net charge of the cell membrane through reducing the amount of alanyl-ester leads to increased binding of autolysins and ultimately increases autolysis [27]–[29]. Point mutations in the *mprF* gene were found in *S. aureus* strains that were resistant to daptomycin and defective in autolysis [30]. However, the *mprF2* mutant in *E. faecalis* 12030 did not show increased autolysis compared with the wild-type (data not shown), indicating that the aminoacylation of PG has no effect on autolysis in enterococcus.

Formation of biofilm is frequently associated with virulence [31] and poses a clinical challenge, especially in foreign body infections [32]. Although *E. faecalis* 12030 is already a strong biofilm producer, the *mprF2* deletion increased biofilm formation about 42% compared to the wild type. This may be caused by pleiotropic or compensatory effects, or by down-regulation of specific biofilm regulators [33], but the exact mechanism has not yet been elucidated. In the complemented mutant, gene *mprF2* was sequenced and was found to contain 3 amino acids changes compared to the wild-type, which may be the reason that the complementation of 12030*ΔmprF2* cannot completely restore the phenotype to the level of the wild type. That the complementation of *ΔmprF2* with vector pMSP3535::*mprF2* did only partly restore the lipid content of the mutant compared to the wild type level may be explained by the fact that expression of the gene could not be induced. Vector pMSP3535 contains a strong nisin promoter, which is capable to over-express the gene cloned into the vector when nisin is added. However, nisin is also a cationic antimicrobial peptide that influences by itself the lipid composition of cell membrane (data not shown). Therefore, nisin was not added for the biofilm formation assay leading to only base-line expression of the gene cloned into pMSP3535 (i.e *mprF2*). This could explain the lower lipid contents extracted from 12030*ΔmprF2* pMSP3535::*mprF2* compared to the wild-type.

Extracellular DNA was measured in biofilms to assess whether the increased biofilm formation in the 12030*ΔmprF2* mutant is caused by eDNA, because it has been previously observed that eDNA may serve as an important matrix component of microbial biofilms. For the major autolysins (AtlE of *Staphylococcus epidermidis* and muramidase 2 of *E. faecalis*) several authors confirmed a role in biofilm formation [34]–[37] and Qin et al [34] described eDNA as an integral component during biofilm formation. The measurement of eDNA release confirmed our biofilm results (see Figure 3), i.e. the 12030*ΔmprF2* mutant showed increased biofilm production and increased eDNA release. While eDNA has been shown previously to be primarily a by-product of cell lysis, the 12030*ΔmprF2* mutant was not significantly different regarding autolysis compared to the wild type in our experiments. This suggests that in Enterococcus, similar to the results by Grande [38], accumulation of eDNA may be not caused only by autolysis but also by other, so far unknown mechanisms.

Although biofilm formation is usually considered a virulence factor, there was no effect seen on the pathogenicity of the isogenic mutant compared to the wild type in a mouse bacteremia model. The increased adherence of the mutant to polystyren may rely on different mechanism and therefore does probably not correspond to an increased adherence to eukaryotic cells in the host. Alternatively, compensatory mechanisms (such as the increased susceptibility of the mutant against antimicrobial peptides) may counteract the increased adherence in vivo. Comparing an *mprF* mutant and a *dltA* mutant in a rabbit endocarditis model, Weidenmeier and colleagues observed that the reduction in virulence of the *dltA* mutation was more pronounced than the *mprF* mutation [39]. Using a mouse bacteremia model, we could detect a significant reduction in virulence in the *dltA* mutant [14] but were not able to observe this effect in the *mprF* mutant. We therefore conclude that, contrary to the observations in *S. aureus* [8], [40], *Listeria monocytogenes* [41], *and Mycobacterium tuberculosis* [42], MprF is probably not a major virulence factor in *E. faecalis*.

Previous studies have shown that a *mprF* mutant of *S. aureus* is killed more readily by neutrophils due to increased susceptibility of phagocytosed bacteria to defensins like HNP1-3 or cathelicidin LL-37 that are stored in the azurophilic granules [8], [40]. Our results are not comparable to these studies, because enterococcus is not killed by complement and phagocytes alone [43]. Only the addition of opsonic antibodies, e.g., those against LTA [15] or capsular polysaccharides [44], results in effective killing. The thick layer of polysaccharide material in enterococci [45] may confer additional resistance against neutrophil killing and probably diminishes the specific effects of MprF. Our findings suggest that bactericidal mechanisms other than CAMPs are more important in the killing of enterococci by neutrophils. *Streptococcus pneumoniae*, for example, is killed even by neutrophils that lack HNP1-3, suggesting a minor role of defensins in the killing of this gram-positive pathogen. Instead, the neutrophil serine proteases elastase, cathepsin G, and proteinase 3 are critical for the intracellular killing of *S. pneumoniae* by neutrophils [46]. Hence, nonoxidative mechanisms such as serine proteases or reactive oxidative species may be of greater importance than CAMPs in the killing of *E. faecalis* by neutrophils.

We investigated the role of *mprF2* as a virulence factor in vivo in a mouse bacteremia model. Our results suggest that the expression of aminoacyl-PG does not affect bacterial survival during bloodstream infection. In contrast, impaired aminoacylation of phosphatidylglyercerol in *S. aureus* increases intracellular killing by neutrophils, associated with a reduced bacterial burden during bacteremia and endocarditis [8], [39]. In bloodstream infections, neutrophils are the first line of defense against invading pathogens, and it is therefore not surprising that MprF is an important virulence factor in this model. Since opsonophagocytic killing was not increased in the *E. faecalis mprF2* mutant, it seems plausible that virulence was also not altered during bloodstream infection. However, we cannot exclude the possibility that inactivation of *mprF2* impairs virulence in other modes of infection, e.g., during colonization or biofilm infection. For example, skin expression of the antimicrobial protein RNase 7 has an important role in the protection of human skin against *E. faecium* colonization [47].The function of *mprF1* is not clear from our results or from the data presented by Roy and Ibba [9], [10]; there seems to be no obvious effect on the cell wall lipids, and the 12030*ΔmprF1* mutant has been tested also by OPA, in the mouse sepsis and in the autolysis assay. However, in none of these tests there was a statistically significant difference between this mutant and the wild-type (data not shown). Whether this protein functions as a sensor or regulator for the expression of *mprF2* must be the subject of future studies.

Analysis of insertional mutants in genes *mprF1 (ef0031)* and *mprF2 (ef1027)* in a plasmid-cured *Enterococcus faecalis* V583-derivative strain (VE14089) has been reported by Rigottier-Gois et al. [43]. This study showed that there was no difference in growth kinetics and resistance to antibiotics for the single cross-over mutants SCO *ef0031* and SCO *ef1027* The SCO *ef1027* mutant was killed by PMNs and complement without the addition of serum. In contrast, we observed that *E. faecalis* 12030 Δ*mprF2* was efficiently killed only by a combination of PMNs with complement and specific antibody. Furthermore, the SCO *ef1027* mutant in *E. faecalis* VE14089 showed decreased virulence in a *Galleria mellonella* model while our Δ*mprF2* mutant in *E. faecalis* 12030 was not significantly affected in virulence in a mouse bacteremia model. While the reasons for these differences have not been studied yet, the different virulence model systems and the different strain background may explain these contrasting results.

In conclusion, our data demonstrate that *mprF2* (*EF_1027*) seems to be the only functional aminoacyl-phosphatidylglycerol synthase in *E. faecalis* in the conditions tested by us. It is responsible for synthesis of three distinct amino-PGs, most likely Lys-PG, Ala-PG, and Arg-PG. MprF2 is involved in resistance against CAMPs but is unnecessary for autolysis, killing by neutrophils, or bacteremia in mice.

## Materials and Methods

### Bacterial Strains, Plasmids, and Growth Conditions

The bacterial strains and plasmids used are listed in Table 2. *E*. *faecalis* strain *12030* was grown in 37°C tryptic soy broth (TSB; CASO broth; Merck) or on tryptic soy agar plates (TSA; CASO agar; Merck). When required, erythromycin (100 or 150 µg/ml) or kanamycin (1000 µg/ml) were added. *Escherichia coli* strains were cultured under vigorous shaking at 37°C in Luria-Bertani broth (LB; Merck) with ampicillin (100 µg/ml), kanamycin (50 µg/ml), or erythromycin (100 µg/ml) when required. All antibiotics were purchased from Sigma Chemicals.

**Table 2:** Enterococcal strains and plasmids used in this study.

Strain or plasmid	Characterization	Reference or source
**Strains**		
*E.faecalis* V583	Reference strain, fully sequenced	[11]
*E.faecalis* 12030	Clinical isolate	[21]
*E.faecalis* 12030*ΔmprF1*	*mprF1* (EF_0031) mutant	This study
*E.faecalis* 12303*ΔmprF2*	*mprF2* (EF_1027) mutant	This study
*E.faecalis* 12030*ΔmprF2* CM	Strain complemented with knock in of *mprF2* gene	This study
*E.faecalis* 12030*ΔmprF2/pMSP3535::mprF2*	Strain complemented with *mprF2* gene *by plasmid pMSP*	This study
*E.coli* TOP10 F	Gram-negative cloning host	Invitrogen
**Plsmids**		
pCASPER	Gram-positive, temp-sensitive mutagenesis vector	[57]
pMAD	Gram-positive, temp-sensitive mutagenesis vector	[51]
pMAD - *ΔmprF1*	pMAD carrying *mprF1* deleted	This study
pCASPER - *ΔmprF2*	pCAPER carrying *mprF2* deleted	This study
pMSP3535	Emr, pAMb1 and ColE1 replicons, *nis*RK, P*nis*A	[54]
pMSP3535::*mprF2*	Expression vector carrying the *mprF2* gene	This study
pCRII-TOPO	Gram-negative cloning vector	Invitrogen

### General Molecular Techniques

Chromosomal DNA from enterococci was prepared using the DNeasy Tissue kit (Qiagen) according to the manufacturer’s instructions. Plasmids were purified using the Wizard Plus SV Miniprep System (Promega). PCR was carried out in a reaction volume of 25 µl with about 100 ng of chromosomal DNA of *E. faecalis* 12030 and Platinum Taq DNA polymerase (Invitrogen); the annealing temperature depended on the calculated melting temperature of primers. In general, 30 cycles were performed, and PCR products were subsequently purified using the QIAquick PCR purification Kit (Qiagen Hilden, Germany). Primers used for this study are listed in Table 3. Custom primers were manufactured by Invitrogen and Sigma. Restriction and modifying enzymes were obtained from New England Biolabs and Fermentas. Electrocompetent enterococci were prepared as described previously [48]. All the other methods (DNA ligation, eletrophoresis, and transformation of competent *E. coli*) used standard techniques [49].

**Table 3:** Primers used in this study.

No.	Name	Sequence (5′–3′) a
1	EF0031DMF EcoRI	CTGTCGAATTCCATCAGCGCTTAGGAATAATTG
2	EF0031DMR SmaI	CTGTCCCCGGGCAACATAACGTAGCCAAAGAG
3	EF0031 inside 1	CAATAATTTAACGACTACATAGTC
4	EF0031 inside 2	GTCACTAGTTGGCAACCAC
5	EF1027 delF	CAGCAATTGGGTTTCTTTGAA
6	EF1027 delR	TTTGATGAGATTCCGCTATGG
7	EF1027 OEL	ACTAGCGCGGCCGCTTGCTCC CCAAGTTGGTGAGTTTCCAGA
8	EF1027 OER	GGAGCAAGCGGCCGCGCTAGT AGCAATCCCAATAATCGAAGC
9	pMSP1027 BamHI	CCTGTCGGATCCGGAAATGAAGGTGTCTAAATGAA
10	pMSP1027 PstI	CCTGTCCTGCAGAATTGAGCTTCTTTTTGTTAGTC

a Linkers are underlined.

### Construction of Deletion Mutants Delta *mprF1* and *mprF2*

A non-polar deletion mutant 12030*ΔmprF1* was constructed. A part of gene *mprF1* (*EF_0031* in *E. faecalis* V583; GenBank accession no. NP_813841) was deleted from aminoacid 84 to 817, i.e. a total of 733 aa was deleted using the method described by Le Jeune et al. [50] with the following modification. Briefly, two fragments of approximately 900 bp, corresponding to the flanking regions of the target gene, were amplified by PCR using primers shown in Table 3. The DNA fragments were purified, digested with restriction enzymes, and ligated into vector pMAD [51] (Table 2).

A deletion mutant of *mprF2* (*EF_1027* in the *E. faecalis* V583 genome) was created using the method described by Cieslewicz et al. [52]. Briefly, two fragments of approximately 600 bp, corresponding to the flanking regions of the target gene, were amplified by PCR using primers shown in Table 3. This resulted in the deletion of a fragment from nucleotide position 738 to 1538, i.e. 800 bp were deleted. The resulting fragments were cloned into the gram-negative cloning vector pCRII-TOPO (Invitrogen) and excised with restriction enzyme EcoRI.

The *mprF2* fragment was inserted into the gram-positive vector pCASPER (Table 2), which contains a temperature-sensitive origin of replication [53].

The ligation mixtures of the two plasmids pMAD::*ΔmprF1* and pCASPER::*ΔmprF2* were transformed by electroporation into *E. coli* Top10F cells. After selection and verification, the generated recombinant plasmids were used to transform electro-competent *E. faecalis* 12030 cells [14], and gene replacement was performed via double cross-over as described previously [14], [50].

### Complementation of the Deletion Mutants

The entire *mprF2* with flanking regions was amplified using primer-pairs 5/6 (Table 3). The PCR product was cloned into pMAD, and the resulting plasmid pMAD::*mprF2* was transformed into *E. faecalis* 12030*ΔmprF2* by electroporation. Double cross-over and selection of mutants was performed subsequently as described above (knock-in mutant).

The *mprF2* gene was also cloned into expression vector pMSP3535 (Table 2) [54] using primers 9 and 10 to amplify the entire gene *mprF2* including the RBS, start codon (ATG), and stop codon (TGA). The PCR product and plasmid pMSP3535 were digested with restriction enzyme BamHI and PstI and ligated using T4 DNA ligase. The chimeric plasmid *pMSP3535::mprF2* was transformed in *E. coli* Top10, and correct inserts were confirmed by PCR. Plasmid *pMSP3535::mprF2* was extracted from *E. coli* and transformed into *E. faecalis* 12030*ΔmprF2* by electroporation; transformants were selected on TSA plates containing erythromycin.

### Growth Kinetics

Growth curves of the wild-type *E. faecalis* 12030, its isogenic derivative mutants, and the complemented mutants were compared in TSB. An overnight culture was diluted 1∶50 and incubated at 37°C, while the OD_600_ was measured every hour.

### Membrane Lipid Extraction

Lipids were extracted from *E. faecalis* 12030, its isogenic derivative mutants, as well as the complemented mutants using a modified Bligh-Dyer method [55]. For isolation of these strains’ membrane lipids, 500 ml overnight TSB culture grown at 37°C was used. Cultures were cooled on ice for 30 to 60 min, and bacteria were collected by centrifugation and washed with 0.1 M citrate buffer (pH 4.7). The bacterial suspension was mixed with an equal volume of glass beads, and bacterial cells were lysed with a bead beater (Biospec Products, Inc.) by vigorous shaking for 2 min three times at 4°C. Bacteria were cooled on ice between each run for 5 min. Glass beads were sedimented by centrifugation for 1 min at 200×*g*, and bacterial cell membranes were removed with the supernatant. The remaining bacterial debris was again sedimented by centrifugation at 12,000×*g* for 20 min. The pellets were washed with 40 ml of 0.1 M citrate buffer (pH 4.7), the wet weight was determined, and samples were stored frozen at –20°C. For lipid extraction, frozen pellets were resuspended in 0.1 M citrate buffer (pH 4.7). Chloroform and methanol were added to obtain a final chloroform/methanol/buffer ratio of 2∶1∶0.8. Lipids were extracted for 2 h at room temperature with vigorous vortexing. Insoluble material was removed by centrifugation at 2,600×*g* for 20 min, and the extracted lipids were transferred with the supernatant into new tubes. The extraction was repeated as described above; chloroform and buffer were added to the combined extracts to obtain a methanol/chloroform/buffer ratio of 1∶1∶0.8. Following vigorous vortexing, samples were centrifuged at 2,600×*g* for 20 min, and the chloroform phase containing lipids was transferred to a new tube. Lipids were dried under a stream of nitrogen, and the dry weight was determined. Lipids were then resuspended in methanol-chloroform (1∶1) at a concentration of 10 mg/ml and stored at –20°C, and 100-µg samples were analyzed by thin-layer chromatography (TLC).

### Lipid Analysis by TLC

Lipids were separated by TLC using silica 60 F254 HPTLC plates (Merck) and developed with chloroform/methanol/water (65∶15∶2, by volume) in the first direction and chloroform/methanol/acetic acid/water (80∶12∶15∶4, by volume) in the second direction. For detection of phospholipids, TLC plates were stained with molybdenum blue, and aminophospholipids were stained with ninhydrin, as previously described [8]. Phosphatidylgylcerol (PG), diphosphaditylglycerol (DPG), diglycosyldiacylglycerol (DGlcDAG), and monoglycosyldiacylglycerol (MGlcDAG) were identified by commercial (purchased from Sigma-Aldrich) or internal laboratory standards [48] and used to determine the position of the PG and DPG spots in 2D-TLC.

### Biofilm Assay

Biofilm formation was measured as described by Baldassarri et al. [52]. In brief 180 µl TSB supplemented with 1% glucose in a 96 well tissue culture plate (Brand) were inoculated with 20 µl of a stationary phase culture of the respective strain. Afterwards, the plate was incubated for 18 h at 37°C without shaking. To prevent the cultures from drying, the growth environment was kept humid. Bacterial growth was determined the next day by maesuring the OD of each well in a plate reader at a wavelength of 600 nm. After discarding the growth medium and washing the wells three times with PBS, the biofilm was dried for 1 h at 60°C. Subsequently, the biofilm was stained with 100 µl Huckers Crystall Violet for 2–3 min followed by washing the plate under tap water and drying of the stained biofilm. The OD_600_ was measured in a plate reader and the biofilm index was calculated as follows: (OD(Biofilm)×0.5)/OD (Growth).

### eDNA Assay

Analysis of eDNA was carried out as described previously [36], [37]. All strains were cultivated overnight in TSB at 37°C. The culture was diluted 1∶10 in TSB with 1% glucose, and 200 µl of this cell suspension was used to inoculate a sterile black 96-well FIA- plates (Greiner Bio-one). Each strain was cultivated in triplicate. After 18 h at 37°C, wells were gently washed three times with 200 µl of phosphate-buffered saline (PBS), and dried at 60°C for 1 hour. 100 µl of DNA-specific dye SYTOX green (Invitrogen) was added to these wells at a final concentration of 1 µM in DMSO, and incubated for 10 minutes before being spectrofluorometrically measured with excitation wavelength at 485 nm and emission wavelength at 535 nm.

### Triton X-100-induced Autolysis Assays Under Non-growing Conditions

Strains were grown to an OD_600_ of 0.8 in TSB medium and treated as previously described [56]. Briefly, cells were pelleted by centrifugation (4000 g, 10 min at 4°C), washed in the same volume of ice-cold sterile water, and resuspended in the same volume of 50 mM Tris-HCl, pH 7.5, containing 0.1% Triton X-100. The cell suspensions were then transferred into 96-well sterile microplates and incubated at 37°C without shaking. Autolysis was monitored by measuring OD_600_ every 10 min with an automated incubator/optical density reader (Model 680, Bio-Rad Laboratories). The results were normalized to the OD_600_ at time zero, i.e., percent lysis at time t = [(OD at time zero – OD at time t)/OD at time zero]×100.

### Antimicrobial Susceptibility to Polymyxin B, Nisin, Colistin, HBD-3, and Daptomycin

The minimal inhibitory concentration (MIC) of polymyxin B, nisin, HBD-3, and colistin was determined in serial dilution with a modified NCCLS method [29]. Experiments were performed in TSB broth in 96-well microtiter tissue culture plates (Greiner). Wells were inoculated with 50-µl volumes of a suspension containing approximately 1×10^6^ CFU/ml of the test organism, and concentrations were confirmed by enumeration of CFUs after serial dilution. Microtiter plates were incubated overnight at 37°C, and the MICs were defined as the lowest concentration at which visible growth was inhibited. The MIC against daptomycin was determined by E-test according to standard laboratory procedures.

### Opsonophagocytic Assay

The opsonophagocytic assay was performed as described elsewhere [48] using baby rabbit serum as complement source and rabbit sera raised against purified LTA from *E. faecalis* 12030. Polymorphonuclear neutrophils (PMN) were freshly prepared from human blood collected from healthy adult volunteers. The institutional review board of the University of Freiburg approved the study protocol, and written informed consent was obtained from all study participants. Bacterial strains were grown to mid-log phase in TSB, adjusted to 2×10^7^/ml, and mixed with equal volumes of serum (dilution 1∶100), absorbed baby rabbit sera as complement source (dilution 1∶15), and PMNs (adjusted to 2×10^7^/ml). Controls included tubes from which PMNs (PMN^neg^), complement (c’^neg^), or antibody (Ab^neg^) was omitted. The mixture was incubated on a rotor rack at 37°C for 90 min, and samples were plated in duplicate at time 0 and after 90 min. Percent killing was calculated by comparing the colony counts at 90 min (*t*90) of a control not containing PMNs (PMN^neg^) to the colony counts of a tube that contained all four components of the assay using the following formula: {[(mean CFU PMN^neg^ at *t*90) - (mean CFU at *t*90)]/(mean CFU PMN^neg^ at *t*90)}×100.

### Animal Studies

The virulence of *E. faecalis* 12030, its isogenic derivative mutants, and the complemented mutants was evaluated in a mouse bacteremia model [48]. In summary, eight female BALB/c mice 6–8 weeks old were challenged by i.v. injection with 1.5×10^8^ cfu of *E. faecalis* 12030, 12030*ΔmprF2*, and 12030*ΔmprF2* CM (knock-in) via the tail vein. Forty-eight hours after injection the animals were sacrificed, and livers, spleens, and kidneys were removed to assess bacterial loads.

### Ethics Statement

All animal experiments were performed in compliance with the German animal protection law (TierSchG). Mice were housed and handled in accordance with good animal practice as defined by FELASA and the national animal welfare body GV-SOLAS. The animal welfare committees of the University of Freiburg (Regierungspräsidium Freiburg Az 35/9185.81/G-07/15) approved all animal experiments. The institutional review board of the University of Freiburg approved the study protocol, and written informed consent was obtained from all study participants.
